# Simultaneous measurement of passage through the restriction point and MCM loading in single cells

**DOI:** 10.1093/nar/gkv744

**Published:** 2015-08-06

**Authors:** T. W. Håland, E. Boye, T. Stokke, B. Grallert, R. G. Syljuåsen

**Affiliations:** 1 Department of Radiation Biology, Institute for Cancer Research, Oslo University Hospital, Montebello, 0310 Oslo, Norway; 2 Department of Biosciences, Faculty of Mathematics and Natural Sciences, University of Oslo, 0316 Oslo, Norway

## Abstract

Passage through the Retinoblastoma protein (RB1)-dependent restriction point and the loading of minichromosome maintenance proteins (MCMs) are two crucial events in G1-phase that help maintain genome integrity. Deregulation of these processes can cause uncontrolled proliferation and cancer development. Both events have been extensively characterized individually, but their relative timing and inter-dependence remain less clear. Here, we describe a novel method to simultaneously measure MCM loading and passage through the restriction point. We exploit that the RB1 protein is anchored in G1-phase but is released when hyper-phosphorylated at the restriction point. After extracting cells with salt and detergent before fixation we can simultaneously measure, by flow cytometry, the loading of MCMs onto chromatin and RB1 binding to determine the order of the two events in individual cells. We have used this method to examine the relative timing of the two events in human cells. Whereas in BJ fibroblasts released from G0-phase MCM loading started mainly after the restriction point, in a significant fraction of exponentially growing BJ and U2OS osteosarcoma cells MCMs were loaded in G1-phase with RB1 anchored, demonstrating that MCM loading can also start before the restriction point. These results were supported by measurements in synchronized U2OS cells.

## INTRODUCTION

Cancer cells are often deficient in the control of G1-phase and therefore knowledge about the major regulatory events in G1-phase is important for our understanding of carcinogenesis. Two events in G1 are the formation of the pre-replicative complex (pre-RC) and passage through the Retinoblastoma protein (RB1)-dependent restriction point. RB1 was the first tumor suppressor discovered (1) and abnormal levels of pre-RC components can cause DNA damage and genomic instability (reviewed in 2).

Formation of the pre-RC, culminating in the loading of the six minichromosome maintenance (MCM) proteins, is one of the first steps in preparation for DNA replication. Pre-RCs form in G1-phase through a multistep process called licensing: CDC6 is recruited to the origin recognition complex (ORC) after exit from mitosis (3,4). Subsequently, CDT1 and MCM2–7, the replicative DNA helicase, form a complex and are recruited by CDC6 to the ORC to form the pre-RC (5,6). Adenosine triphosphate bound to CDC6 and ORC undergoes hydrolysis, leading to the release of CDT1 and CDC6 and to the loading of MCM2–7 helicases onto DNA (6–8). A chain of events including phosphorylation by CDC7, recruitment of CDC45, further phosphorylations by cyclin-dependent kinases (CDKs) and recruitment of several additional replication factors activate the helicase and DNA replication is initiated (9,10). Once the cells enter S-phase several of the licensing factors are degraded or inhibited, ensuring that no origin can be relicensed after replication has commenced (11–14). In this way the formation and dissociation of the pre-RCs help ensuring that the DNA is replicated once and only once per cell cycle.

The restriction point was first described in 1974 as a specific time point in G1-phase when the cell becomes committed to another round in the cell cycle (15). Over the last four decades the restriction point has been investigated extensively, often focusing on the phosphorylation status of RB1 (1,16,17). RB1 is phosphorylated early in G1 by CDK4/6-cyclinD (18,19). The common view was that increasing levels of RB1-phosphorylation by CDK4/6-cyclin D through G1 leads to a partial release of the E2F transcription factor from its RB1-bound form, thereby enabling transcription of E2F target genes, allowing passage through the restriction point (reviewed in 20). However, recent work has shown that CDK4/6-cyclin D can only mono-phosphorylate RB1 and this phosphorylation activates rather than inactivates RB1, stimulating its binding to E2F and thus inhibiting transcription of E2F target genes (19,20). As G1-phase progresses, the CDK2–cyclinE complex inactivates RB1 by further phosphorylating the protein and this phosphorylation is considered a molecular marker for the restriction point (21). In this hyper-phosphorylated state, past the restriction point, RB1 can no longer bind E2F. Free E2F can translocate into the nucleus and stimulate transcription of target genes (22), several of which are involved in DNA replication initiation. Notably, many pre-RC components, such as MCM2–7, CDT1 and CDC6 have E2F binding sites in their promoter (23–25), leading to the idea that RB1 hyper-phosphorylation is likely to precede the loading of MCMs. However, even though both the restriction point and MCM loading have been extensively studied separately, the relative timing of these processes and their inter-dependence remain less clear.

Here we have developed a novel method that enables us to simultaneously study MCM loading and RB1 hyper-phosphorylation in single cells. By this method we can assess the relative timing of the two events and thereby address whether or not these events are connected and, if so, in which order they appear. The results show that while MCMs are often loaded after RB1 hyper-phosphorylation, they can in many cases be loaded prior to RB1 hyper-phosphorylation.

## MATERIALS AND METHODS

### Cell culture and synchronization

Human BJ fibroblast and human U2OS osteosarcoma cells were cultivated in Dulbecco's modified Eagle's Medium (DMEM) (Invitrogen) supplemented with 10% fetal bovine serum and 1% Penicillin/Streptomycin at 37°C in a humidified environment with 5% CO_2_. Arrest of BJ cells in G0-phase was achieved by growing the cells to 100% confluence followed by addition of fresh culture medium and subsequent incubation for three additional days. For release of cells from G0-phase, the cells were subcultured at low density. For synchronization with Nocodazole (Sigma-Aldrich) the drug was present at 0.04 μg/ml for 12 h before mitotic cells were collected by manual shaking and washed three times in phosphate buffered saline (PBS) and incubated in regular medium. Irradiation was performed using an X-ray generator (Faxitron CP160, 160kV, 6,3mA, 1Gy/min).

### Extraction and fixation

To extract unattached proteins for flow cytometry analysis, the medium was removed and cells were washed once with PBS, followed by a quick rinse in 1 ml trypsin and incubation for 2 min at 37°C to detach the cells. The cells were resuspended in DMEM and collected by centrifugation, and the cell pellet was treated with 750 μl low salt extraction buffer (0.1% Igepal CA-630, 10 mM NaCl, 5 mM MgCl_2_, 0.1 mM PMSF, 10 mM Potassium phosphate buffer (pH 7.4)) for 5 min on ice. Then the cells were fixed by adding 250 μl of extraction buffer containing 10% formalin (HT501128 SIGMA) (final concentration 2.5%) and incubation was continued for 1 h on ice. The cells were washed with PBS and stored at 4°C for up to 24 h before staining with antibodies. Unextracted cells were fixed in 2.5% formalin for 1 h on ice before washing with PBS and permeabilization in 1 ml 70% ethanol at −20°C. To extract unattached proteins for immunoblotting, cells were incubated 5 min on ice with 1 ml low salt extraction buffer prior to cell lysis.

### Immunoblots

Whole-cell lysates were prepared in 2× Laemmli sample buffer. Proteins were separated by Sodium dodecyl sulphate-polyacrylamide gel electrophoresis, on 7% gels (26). The proteins were transferred to a PVDF membrane in a wet transfer cell (Bio-Rad) using a buffer containing 25 mM Trizma base and 192 mM Glycine. After transfer the proteins were fixed to the membrane in 100% methanol for 5 min. The membranes were blocked in 5% non-fat milk before probing with the relevant antibody: γ-tubulin, diluted 1:10 000 (Sigma-Aldrich T6557), RB1 1:500 (BD Biosciences 554136), RB1-phospho (ser795) 1:1000 (Abcam ab47474). Detection was performed using an enhanced chemiluminescence kit (Amersham Biosciences) and quantified in a Chemi Genius (Syngene) with the software GeneTools.

### Flow cytometry

After fixation the samples were incubated with primary antibodies (RB1 1:50 (BD Biosciences 554136, MCM3 1:100 (N-19, sc-9850)) and secondary antibodies (Alexa Flour 488 and 647) diluted 1:1000 in flow buffer (0.1% Igepal CA-630, 6.5 mM Na2HPO4, 1.5 mM KH2PO4, 2.7 mM KCl, 137 mM NaCl, 0.5 mM ethylenediaminetetraacetic acid (pH7.5)), containing 4% non-fat milk and stained with the DNA-stain Hoechst 33258 (1.5 μg/ml). A barcoding flow cytometry technique was used to eliminate antibody staining variations between individual samples in the ionizing irradiation experiment (Figure 3 and Supplementary Figure S2). Four fixed samples were stained with different concentrations (0.125, 0.031, 0.0062 and 0.00078 ng/μl) of Pacific Blue (Invitrogen) for 30 min in the dark at room temperature and subsequently mixed into one tube. The mixed cells were then stained as above, except that the secondary antibodies were anti-goat Alexa Fluor 488 (1:1000) and anti-mouse Dylight 549 (1:500), and the DNA-stain was FxCycle Far Red (Life Technology). The samples were analyzed on an LSRII flow cytometer (BD Biosciences) using Diva or FlowJo software. All experiments were performed three times or more.

## RESULTS

### Simultaneous measurements of MCM loading and RB1 binding

**Figure 1.**
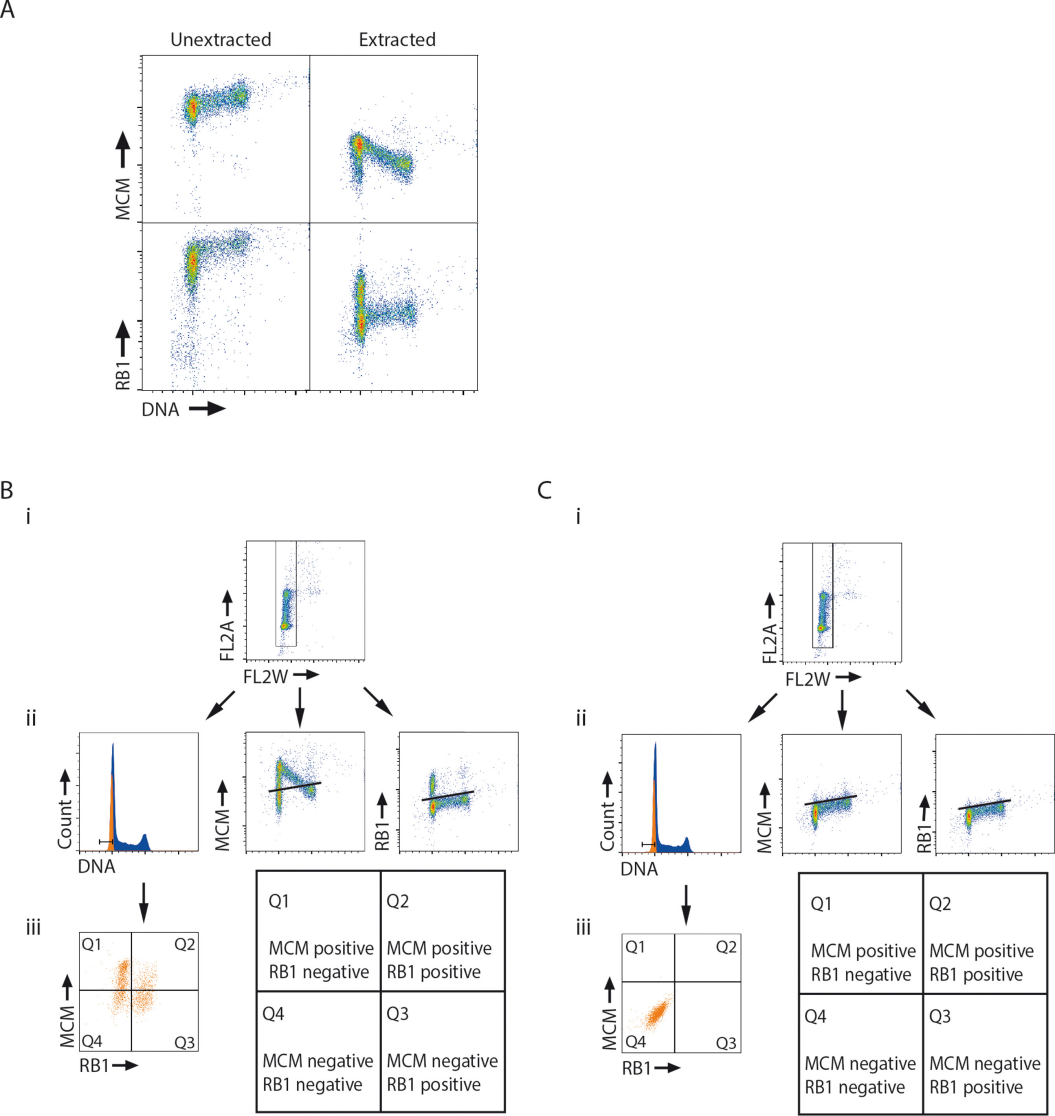
Basic experimental approach. (**A**) Flow cytometric analysis of U2OS cells pre-extracted with salt and detergent before fixation (extracted) or fixed without pre-extraction (unextracted). Cells were stained with antibodies against MCM3 (MCM) and RB1 (RB1) and the DNA-stain Hoechst (DNA). Density scatter plots are shown for MCM staining versus DNA content (top) and RB1 staining versus DNA content (bottom). (**B**) and (**C**) (i) Gating for single cells based on the area (FL2A) and the width (FL2W) of the signal for DNA staining. (ii) Gate defining G1-phase (left), regions defining MCM-positive cells (middle) and cells with RB1 anchored (right). (iii) Scatter plot of MCM versus RB1 for the gated G1 cell population with quadrants indicating the MCM- and RB1-positive and negative cells defined as in B (left), A schematic explanation of quadrants (right). In (C) the samples were stained with secondary antibodies only.

To understand the relationship between pre-RC loading and the restriction point we developed a method to measure both MCM loading and RB1 hyper-phosphorylation in individual cells by flow cytometry. MCMs are in vast excess over replication origins in the cell and, in each cell cycle, only a fraction of them becomes part of pre-RCs and bound to chromatin (27). In order to remove unbound MCMs the cells were extracted before fixation (28) and to measure loading of the MCM complex we used an antibody to MCM3 (see ‘Materials and Methods’ section). Unextracted U2OS cells showed high levels of MCMs in all cell-cycle phases, whereas extraction reduced the MCM signal and gave a distinct pattern of MCM-positive cells (Figure 1A, top). The pattern of MCM-positive cells showed increasing amounts of chromatin-bound MCMs in G1-phase, followed by a decrease during S-phase. This pattern is in agreement with the interpretation that pre-RCs form during G1-phase and the MCMs are offloaded as the replication forks meet during S-phase (29,30). We conclude that our extraction procedure removes the MCM proteins not bound to chromatin, leaving for detection only the fraction of MCM proteins bound in pre-RC complexes.

Previous studies have shown that hypo-phosphorylated RB1 is anchored to the nucleus of G1-phase cells (31), whereas hyper-phosphorylated RB1, occurring past the restriction point, is not bound (28). Thus, we reasoned that when cells are pre-extracted with low salt buffer before fixation, as described above, only G1-phase cells prior to the restriction point retain bound RB1. After the restriction point, RB1 is hyper-phosphorylated, released from its anchor and is washed out during the extraction (28). The cells analyzed for MCM binding were co-stained with an antibody against RB1. Like we found for the MCMs, there were high levels of RB1 in all cell-cycle phases in the unextracted samples (Figure 1A bottom). However, after extraction only some of the G1-phase cells were RB1-positive, most likely representing cells prior to the restriction point. Cells in S-phase showed a clear drop in the RB1 signal compared to the RB1-positive population in G1-phase (Figure 1A bottom), in agreement with S-phase cells containing hyper-phosphorylated and unbound RB1. Thus, flow cytometry using low-salt extraction can be used to detect the RB1 phosphorylation status in single cells under conditions in which bound MCMs can be detected simultaneously.

We then defined appropriate regions and gates to quantify the results. First, single cells were gated based on the width (FL2W) and area (FL2A) of the signal from DNA staining, to exclude multimers of cells (Figure 1B (i)). Next, regions were set to identify the MCM- and RB1-positive cells. MCM-positive cells were defined as those with an MCM-specific signal exceeding that obtained from cells at the S/G2 border, where MCM offloading is presumed to be complete. It should be noted that MCM loading takes place during a time window in G1. MCM-positive cells include all cells that have started to load, regardless of where in this time window they are or how many of their replication origins have assembled pre-RCs. RB1-positive cells were defined based on the difference between the positive cells in G1-phase and the negative cells in S-phase (Figure 1B (ii)). These regions are located at the same place as regions set based on the secondary antibody control (Figure 1C (ii)). Finally, G1-phase cells were gated based on the DNA histogram obtained from the Hoechst signal. To minimize the number of S-phase cells in the gated G1-population, only cells appearing in the left side of the G1 peak (Figure 1B (ii)) were included in the gate. These gates and regions were then used to define the MCM/RB1 status of the G1-cells (Figure 1 (iii)). The G1-cells were displayed in a two-parametric (MCM versus RB1) plot and four quadrants were defined: MCM-positive and RB1-negative (MCM+/RB1−) are in quadrant 1 (Q1), MCM-positive and RB1-positive (MCM+/RB1+) in quadrant 2 (Q2), MCM-negative and RB1-positive (MCM−/RB1+) in quadrant 3 (Q3) and MCM-negative and RB1-negative (MCM−/RB1−) in quadrant 4 (Q4). Thus, the cells in Q2 and Q3 are before the restriction point, with hypo-phosphorylated, anchored RB1 and with MCMs loaded (Q2) or not loaded (Q3). The cells in Q1 are after the restriction point with hyper-phosphorylated RB1 and loaded MCMs. However, the cells in Q4 may either be after the restriction point with hyper-phosphorylated RB1 and the MCMs not yet loaded, or before the restriction point very early in G1 with RB1 not yet hypo-phosphorylated and the MCMs not loaded.

### Validation of the method: BJ cells released from stationary phase

**Figure 2.**
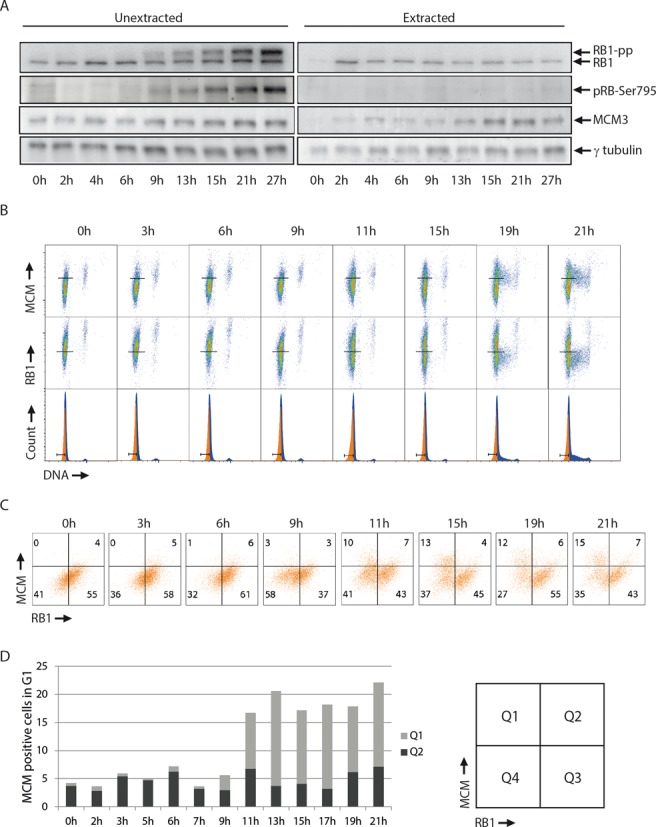
Validation of the method in BJ cells released from G0-phase. (**A**) Immunoblot analysis of BJ cells released from G0-phase after synchronization by contact inhibition. Cells were pre-extracted with salt and detergent (extracted) or left unextracted (unextracted) before cell lysis and processed for immunoblotting with antibodies against total RB1 (RB1), phosphorylated RB1 on Ser795 (pRB1-Ser795) and γ-tubulin. RB1-pp indicates a hyper-phoshorylated form of RB1 detected by the RB1 antibody. Time points indicate hours after release from G0-phase. Note that the blot shown for pRB-Ser795 was from a separate gel than the others, and the corresponding γ-tubulin blot is shown in Supplementary Figure S1A. (**B**) BJ cells were synchronized and released as in (A) and analyzed by flow cytometry as in Figure 1B, at the indicated times after release. Lines in the density scatter plots (top and middle rows) indicate regions defining positive and negative cells for either MCM or RB1. Gates for G1-cells are indicated in the DNA histograms (bottom). (**C**) Scatter plots of MCM versus RB1 for the gated G1 cell population with quadrants indicating the MCM- and RB1-positive and negative cells defined as in (B). Numbers indicate the percentages of G1 cells in each quadrant. (**D**) Column charts showing the percentages of MCM-positive G1 cells in the scatter plots shown in (C) (quadrants Q1 and Q2). Black bars (Q2) indicate cells with RB1 anchored (before restriction point) and gray bars (Q1) cells with RB1 hyper-phosphorylated and no longer anchored (after restriction point). Note that cells start passing the restriction point from 9 hours (RB1-pp in A and two RB1 populations in (C) from 9 h) and S-phase cells are visible from about 15 h (S-phase pattern of MCM in the 15 h MCM plot in (B)). See Supplementary Figure S1B for EdU incorporation versus MCM loading from a similar experiment.

We used the above experimental setup to investigate the loading of MCMs and the RB1 phosphorylation status in normal fibroblasts (BJ) released from G0-phase. BJ cells were synchronized in G0-phase by contact inhibition, released and analyzed by flow cytometry at different time points until they entered S-phase. The RB1 phosphorylation status was also assessed by immunoblotting, showing that hyper-phosphorylated RB1 appeared about 9 h after release from G0-phase (Figure 2A, left panel and Supplementary Figure S1A). Consistent with only hypo-phosphorylated RB1 being anchored in the cell, the hyper-phosphorylated form of RB1 was not detected in extracted samples (Figure 2A, right panel). Furthermore, flow cytometric analysis of extracted cells showed a distinct population, with low RB1 signal, increasing from about 9 h (Figure 2B and C), correlating with the appearance of hyper-phosphorylated RB1 from the same timepoint, as measured by immunoblotting (Figure 2A). The population containing not-anchored Rb in the early time points and in G0 is most likely cells with RB1 not yet phosphorylated and therefore not yet bound (18–20). We conclude that our flow cytometric analysis of anchored RB1 can be used as a measurement of RB1 phosphorylation status. Therefore, the cells start passing the restriction point about 9 h after release from G0.

MCM loading increased from about 11 h (Figure 2C and D and Supplementary Figure S1B). The increase in the MCM-positive population from around 11 h occurred mainly in Q1 (post-restriction point), while the number of cells in Q2 (pre-restriction point) remained low and fairly constant throughout the experiment (Figure 2D). Since the MCM loading increased exclusively in the Q1 population, the vast majority of BJ cells released from G0-phase appear to load MCMs after the restriction point.

**Figure 3.**
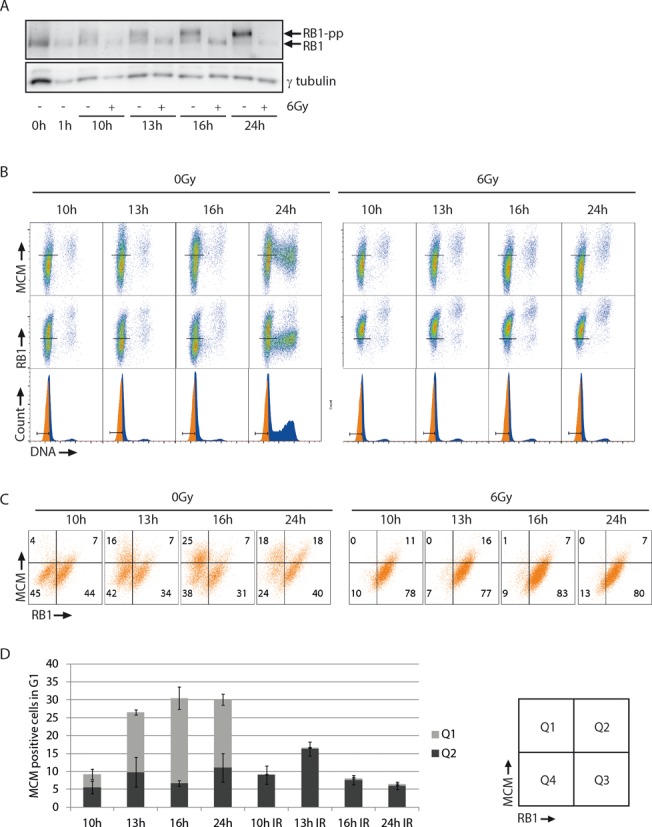
RB1 hyper-phosphorylation and MCM loading after ionizing radiation. (**A**) Immunoblot analysis of BJ cells synchronized and released as in Figure 2A and either treated (+) or not treated (−) with 6 Gy X-ray irradiation at 1 h after release. Cells were processed for immunoblotting similar to the unextracted samples in Figure 2A, with antibodies against total RB1 (RB1) and γ-tubulin. (**B**) BJ cells were synchronized and treated as in (A) and analyzed by flow cytometry as in Figure 2B, except that bar-coding with Pacific Blue was included to minimize sample-to-sample variation. Two non-irradiated and two irradiated samples (e.g. 10 and 13 h) were bar-coded with different concentrations of Pacific Blue and mixed before antibody staining and analysis, allowing the use of identical MCM and RB1 regions for the four samples (Supplementary Figure S1A). The individual samples were thereafter separated by gating on the Pacific Blue-specific signal (Supplementary Figure S1B). (**C**) Scatter plots of MCM versus RB1 for the gated G1 populations in (B). (**D**) Column charts showing the percentages of MCM-positive cells in the scatter plots shown in (B), similar as in Figure 2D. Mean values from three independent experiments are shown. Error bars represent standard error of the mean.

Next, we wanted to check whether our method could detect changes in the kinetics of RB1 phosphorylation and MCM loading after induction of a radiation-induced G1 checkpoint arrest. We treated the BJ cells with ionizing radiation 1 h after release from G0-phase and analyzed the samples by immunoblotting and flow cytometry as above. In addition, we employed bar-coding with Pacific Blue (32,33) to eliminate any sample-to-sample variation in the definition of regions for MCM- and RB1-positive cells (Supplementary Figure S2). Since the unirradiated control cells showed a gradual increase of hyper-phosphorylated RB1 from around 9 h (Figure 2A), we examined the irradiated cells at 10–24 h after release. In agreement with a radiation-induced G1-arrest, immunoblot analysis clearly demonstrated a lack of RB1 hyper-phosphorylation in the irradiated samples at 10–24 h after release (Figure 3A). Consistently, flow cytometric analysis of the irradiated, pre-extracted samples lacked the cell population with low RB1 signal at all time points, meaning that all the irradiated cells were arrested before the restriction point with bound RB1 (Figure 3B and C). In agreement with these findings, the irradiated cells did not enter S-phase during the course of this experiment, as seen by lack of increased DNA content (Figure 3B, bottom row). At all times, the MCM loading was higher in the non-irradiated than in the irradiated cells (Figure 3D). This difference was due to the population of cells in Q1 appearing selectively in the non-irradiated samples, representing cells with loaded MCMs past the restriction point (Figure 3D). We conclude that radiation caused an arrest of the cells prior to the restriction point accompanied by a delay in MCM loading. These results demonstrate that our method can detect radiation-induced changes in RB1 phosphorylation and MCM loading and support the conclusion above that MCM loading occurs after the restriction point in BJ cells released from G0-phase.

### Loading of MCMs before the restriction point in cycling cells

**Figure 4.**
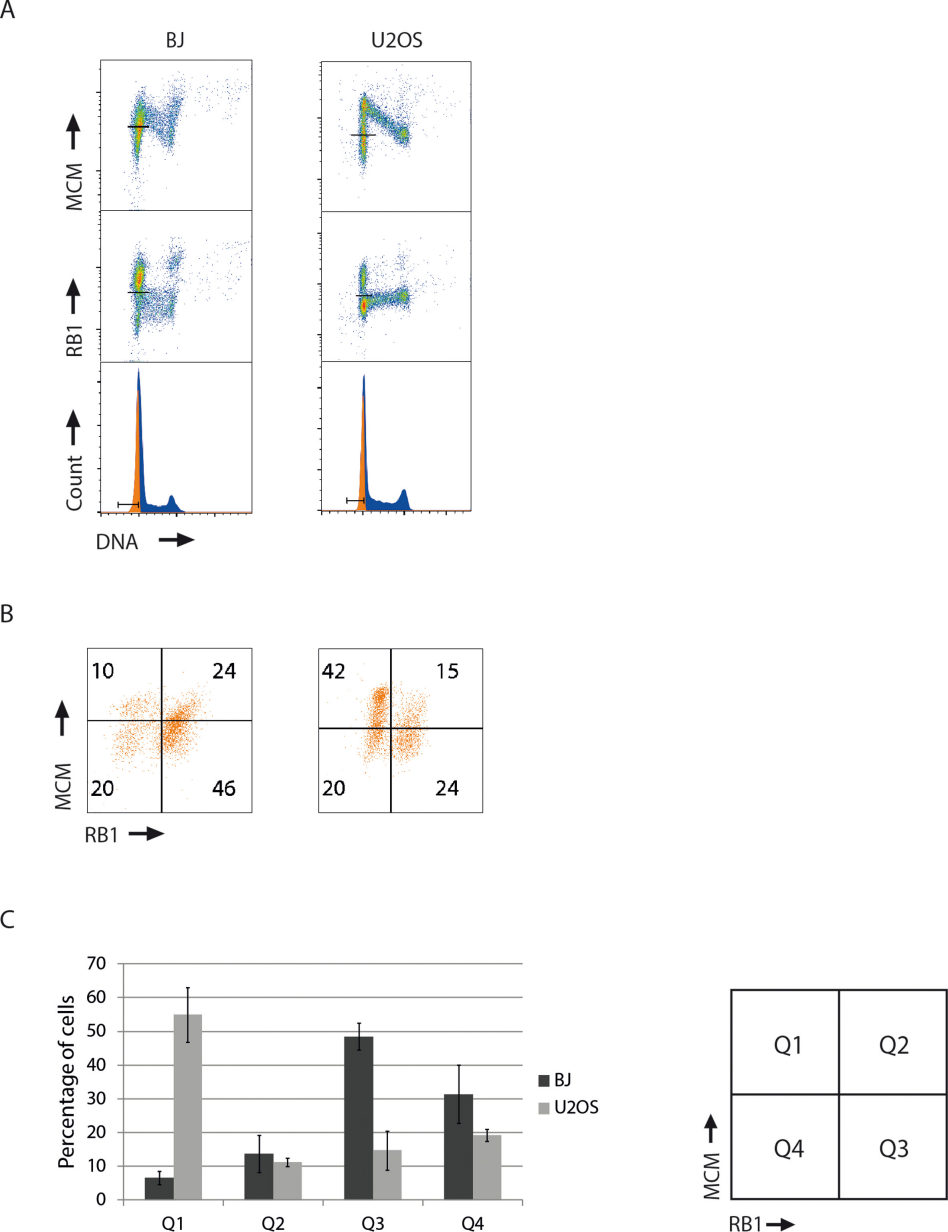
MCMs can be loaded before RB1 hyper-phosphorylation in exponentially growing cells. (**A**) Exponentially growing BJ or U2OS cells were analyzed by flow cytometry for MCMs and RB1 as in Figure 1B. Lines in the density scatter plots (top and middle) indicate the regions defining positive and negative cells for either MCM or RB1. Gates for G1-cells are indicated in the DNA histograms (bottom). (**B**) Scatter plots of MCM versus RB1 for the gated G1 populations in (A). (**C**) Quantification of the percentages of G1 cells in each quadrant from scatter plots as in (B). Mean values from five experiments are shown for BJ (black bars) and U2OS (gray bars) cells. Error bars represent standard error of mean.

We applied the same method to examine the relative timing of MCM loading and RB1 hyper-phosphorylation in unperturbed, exponentially growing BJ and U2OS cells (Figure 4A and B). On average about 14 and 11% of the G1 population of BJ and U2OS cells, respectively, appeared in Q2 (Figure 4C), representing cells that have hypo-phosphorylated RB1 and loaded MCMs. The fraction of cells in Q2 suggests that a sizeable proportion of the G1-cells can load MCMs before RB1 is hyper-phosphorylated. On the other hand, about 7% (BJ) and 55% (U2OS) of the cells in G1-phase appeared in Q1 (after restriction point and with loaded MCMs) and a proportion of these cells most likely load MCMs after RB1 hyper-phosphorylation (Figure 4C). It is interesting to note that the fraction of cells with MCM loaded after the restriction point (Q1) was very different in the two cell lines. This difference likely reflects a longer G1-phase in BJ cells compared to U2OS cells. When G1 is longer, a higher fraction of the G1 cells are before the restriction point, since the length of G1 after the restriction point does not vary much from cell line to cell line (28). Furthermore, a fraction of exponentially growing BJ cells appeared to have loaded MCMs already in G2/M-phase (Figure 4A top left plot, MCM staining in cells with G2/M-phase DNA content). Although we do not know at what point in G2/M-phase these cells loaded MCMs, these results may support previous reports where MCM loading was shown to start in late mitosis (34,35). Alternatively, some of these cells might be post-mitotic binuclear G1 cells that have not completed cytokinesis. Interestingly, the G2/M cells with loaded MCMs contained bound RB1 (Supplementary Figure S3), indicating MCM loading before RB1 hyper-phosphorylation. Altogether, these results suggest that there is no strict order of MCM loading versus RB1 hyper-phosphorylation in exponentially growing BJ or U2OS cells.

### U2OS cells released from Nocodazole block can load MCMs before RB1 hyper-phosphorylation

**Figure 5.**
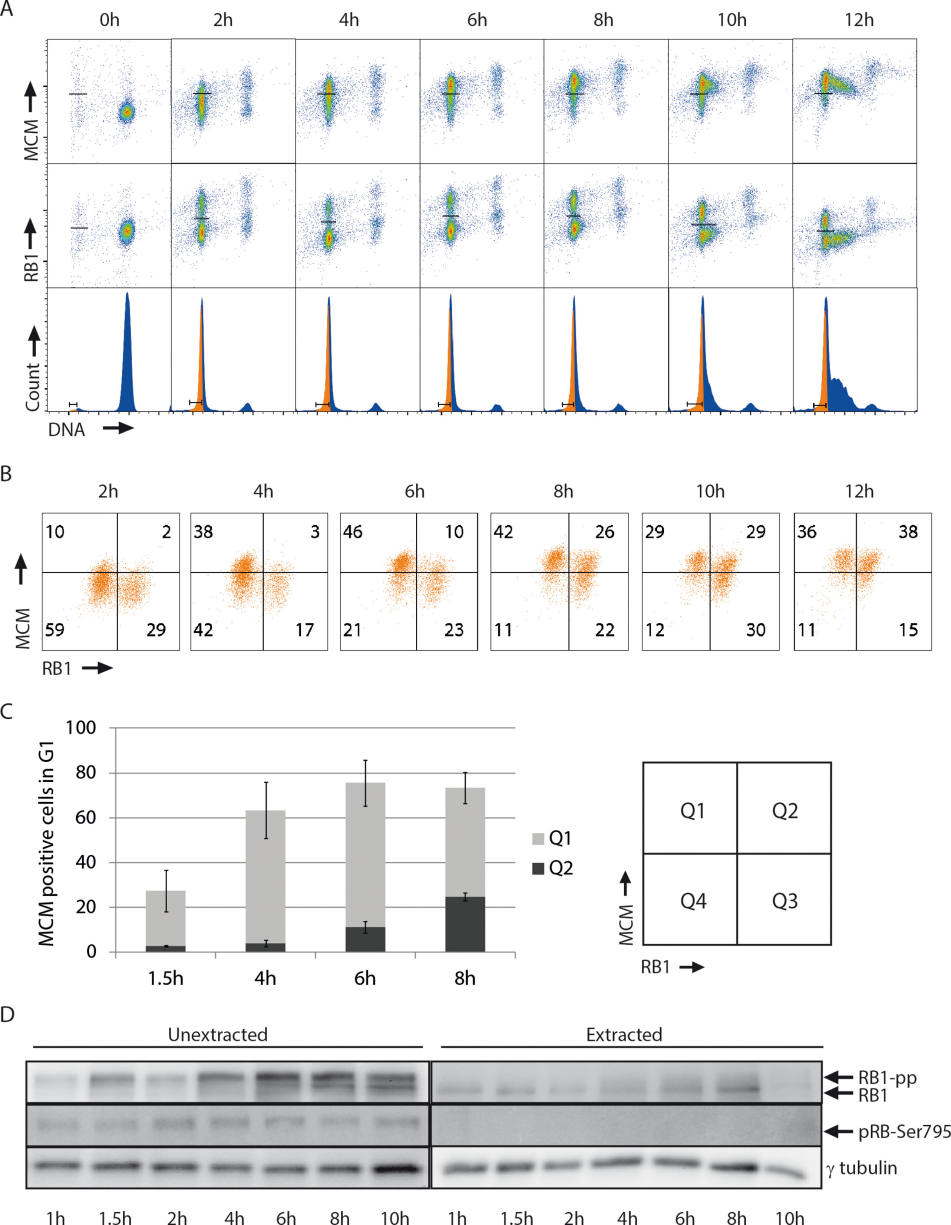
U2OS cells released from Nocodazole block can load MCMs before RB1 hyper-phosphorylation. (**A**) U2OS cells synchronized by release from Nocodazole-arrest were analyzed by flow cytometry for MCMs and RB1 as in Figure 1B. The time points (2–12 h) indicate times after release from the Nocodazole-arrest. Lines in the density scatter plots (top and middle) indicate the regions defining positive and negative cells for either MCM or RB1. Gates for G1-cells are indicated in the DNA histograms (bottom). (**B**) Scatter plots of MCM versus RB1 for the gated G1 populations in (A). (**C**) Column charts showing the percentages of MCM-positive cells (quadrants Q1 and Q2) in the scatter plots shown in (B). Mean values from three independent experiments are shown. Error bars represent standard error of the mean. (**D**) Immunoblot analysis as in Figure 2A of unextracted and extracted U2OS cells. Cells were synchronized and released as in (A). The time points indicate time after release from Nocodazole-arrest.

To further examine the order of RB1 hyper-phosphorylation and MCM loading, U2OS cells were treated with the mitotic inhibitor Nocodazole for 12 h, the mitotic cells were collected and released into the cell cycle, and the status of MCM and RB1 were analyzed as above. Under these conditions the cells started entering S-phase by 10 h after release, as judged by an increase in cellular DNA content (Figure 5A, bottom row). The loading of MCMs started very early in G1-phase and 4 h after release about 40% of the cells had loaded MCMs (Figure 5A–C, 4 h time point, quadrants Q1 + Q2). Immunoblotting showed the presence of hyper-phosphorylated RB1 at all time points (Figure 5D, left panel), and therefore RB1 was hyper-phosphorylated already very early in G1-phase in a large proportion of the cells. However, a subpopulation of cells with hypo-phosphorylated, anchored RB1 was also present at all timepoints, as seen by both flow cytometric analyses (Figure 5A–C, quadrants Q2 + Q3) and by immunoblotting of extracted samples (Figure 5D, right panel, RB1 band). Analyses of MCM versus RB1 in G1-phase cells revealed that the fraction of cells in Q2 increased from 6–8 h after release (Figure 5B and C), meaning G1 cells with loaded MCMs and anchored RB1 before the restriction point. At 8 h about 25% of the cells were in Q2 and this population increased to 38% by 12 h (Figure 5A and B, 8 and 12 h timepoints). Thus, in a proportion of the G1-cells, the MCMs were loaded prior to RB1 hyper-phosphorylation, consistent with our conclusion above that MCM loading can occur both before and after RB1 hyper-phosphorylation. Of note, the fraction of cells in Q2 started to increase at a later time (6–8 h) after mitosis than the fraction of cells in Q1 (1.5–4 h). Thus, two different waves of MCM loading appear to occur after release from Nocodazole arrest. Interestingly, this finding is analogous to those in a recent report suggesting the existence of two populations in cells exiting from mitosis; one with immediate high CDK activity rapidly entering S-phase and the other with low CDK activity entering S-phase several hours later (36).

## DISCUSSION

We have developed a novel method allowing us to study the order of MCM loading and RB1 hyper-phosphorylation in single cells by flow cytometry. In this method we have exploited the fact that hyper-phosphorylation of RB1 at the restriction point is accompanied by a loss of RB1 anchoring in the cell (28). Passage through the restriction point (i.e. RB1 anchoring status) and MCM loading can be simultaneously measured by antibody staining following a common extraction procedure with salt and detergent. The flow-cytometric analysis makes it possible to rapidly assess MCM loading and the phosphorylation status of RB1 in thousands of individual cells. Because we are looking at both parameters simultaneously, this method can be used to address how these two events are coordinated. The method is fairly straightforward and can be utilized to study RB1 hyper-phosphorylation and the loading of MCMs under different conditions and after different stresses, as well as to investigate other proteins anchored in the cell.

Interestingly, our results suggest that a fraction of exponentially growing U2OS and BJ cells and of U2OS cells released from Nocodazole load MCMs before the RB1 protein is hyper-phoshorylated and presumably before the E2F transcription factors are activated (Figures 4 and 5). However, BJ cells released from G0-phase did not appear to load MCMs before RB1 hyper-phosphorylation (Figures 2 and 3). This difference between synchronized and exponentially growing BJ cells is likely due to a delayed MCM loading in the cells released from G0-phase as compared to exponentially growing cells. Factors needed for MCM loading may be more available in cycling cells, making it possible to start MCM loading before RB1 hyper-phosphorylation. However, two aspects of the regulation of CDC6 can induce a delay in pre-RC loading when the cells are arrested in G0. First, arrest in G0-phase strongly reduces CDC6 protein levels and CDC6 needs to be synthesized *de novo* as cells return to G1-phase (37–39). Second, phosphorylation of CDC6 by CDK2/Cyclin E precedes MCM loading in cells released from G0-phase (40), further postponing pre-RC loading.

Our results show that irradiation of BJ cells released from G0-phase significantly delayed RB1 hyper-phosphorylation as well as MCM loading (Figure 3). The observed inhibition of RB1 hyper-phosphorylation after ionizing radiation is consistent with previous studies (41). The delay in loading of MCMs after irradiation may be due to degradation of CDT1 and CDC6 in irradiated human cells (42,43). Whether the delayed hyper-phosphorylation of RB1 and subsequent lack of E2F-mediated transcription of MCMs also contributes to the delayed MCM loading after ionizing radiation is not known and would be an interesting issue for further investigations.

The classic model is that the restriction point coincides with RB1 hyper-phosphorylation which, in turn, leads to E2F activation and induction of transcripts required for S-phase entry, including those required for MCM loading (44). Our finding that MCM loading can occur before RB1 hyper-phosphorylation in a sizeable fraction of exponentially growing cells is apparently in conflict with this model. However, recent reports have indicated that the classic view of the restriction point and regulation of G1/S by E2F needs to be revisited (45–48) and, in particular, that E2F-induced transcripts are not essential for entry into S-phase (48). Furthermore, *in vivo* studies in E2F-knockout mice have shown that E2F proteins are not essential for proliferation, although an increase in DNA damage and apoptosis is observed during such proliferation, consistent with a role in transcriptional control (16,49,50). Our result that MCM loading can occur before RB1 hyper-phosphorylation implies that the release of E2F from RB1 complexes and E2F-driven transcription is not essential for pre-RC formation.

## Supplementary Material

SUPPLEMENTARY DATA
