# Supplementary material for: Simultaneous measurement of passage through the restriction point and MCM loading in single cells
Source: Nucleic Acids Res. 2015 Aug 6;43(22):e150. doi: 10.1093/nar/gkv744 (PMC4678840; doi:10.1093/nar/gkv744)
Supplement: SUPPLEMENTARY DATA [file supp_43_22_e150__index.html]

Simultaneous measurement of passage through the restriction point and MCM loading in single cells — SUPPLEMENTARY DATA 

# Simultaneous measurement of passage through the restriction point and MCM loading in single cells

## SUPPLEMENTARY DATA

- SUPPLEMENTARY DATA
- Figure S1
- Figure S2
- Figure S3
