# Supplementary material for: Simultaneous measurement of passage through the restriction point and MCM loading in single cells
Source: Nucleic Acids Res. 2015 Aug 6;43(22):e150. doi: 10.1093/nar/gkv744 (PMC4678840; doi:10.1093/nar/gkv744)
Supplement: SUPPLEMENTARY DATA [file supp_gkv744_nar-00925-met-g-2015-File007.docx]

Supplementary figures

Figure S1. Cell-cycle analysis of BJ cells released from G0 phase.

(A) Immunoblot analysis of BJ cells as in Figure 2A for pRB-Ser795 with the corresponding γ-tubulin blot. Note that the G0 sample is overloaded. (B) Cells were treated and analyzed as in Figure 2. EdU was added at the time of release. Scatter plots of EdU vs DNA content are shown for the time points indicated (upper row). In the same experiment cells from parallel dishes were harvested and examined for MCM loading (lower two rows).

Figure S2. Barcoding of samples for flow cytomtery.

(A) Four samples (L1-L4) were labeled with different concentrations of Pacific Blue, and collected into one tube before antibody staining and analysis by flow cytometry. The individual samples were subsequently gated based on four distinct populations in the scatter plot of Pacific Blue versus SSC (Side-Scatter-Signal) (B) After gating of each individual sample based on the Pacific Blue Versus SSC signal (left), analysis of DNA content and MCM and RB1 are performed on each individual sample as in Figure 1B.

Figure S3. MCM-positive G2/M cells contain anchored RB1

Exponentially growing BJ cells were harvested and analyzed by flow cytometry as in Figure 4. DNA histograms and scatter plots of RB1 or MCM vs DNA content are shown. G2/M cells displaying high MCM-signal were gated on the MCM vs DNA content plot and are shown in red color also on the RB1 vs DNA content plot.
