# Supplementary figures and images for: Simultaneous measurement of passage through the restriction point and MCM loading in single cells
Source: Nucleic Acids Res. 2015 Aug 6;43(22):e150. doi: 10.1093/nar/gkv744 (PMC4678840; doi:10.1093/nar/gkv744)

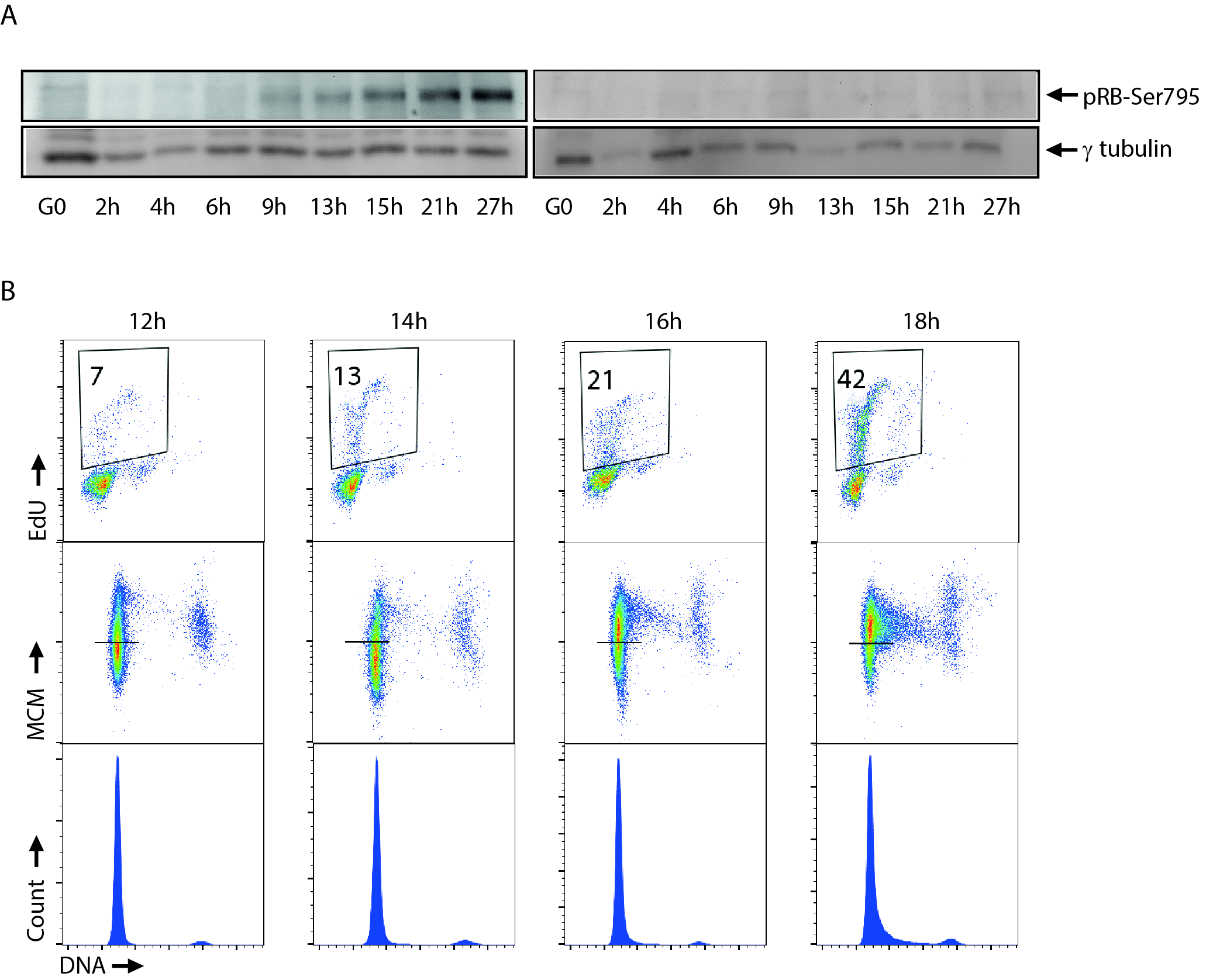

Supplement: SUPPLEMENTARY DATA [file supp_gkv744_nar-00925-met-g-2015-File008.tif]

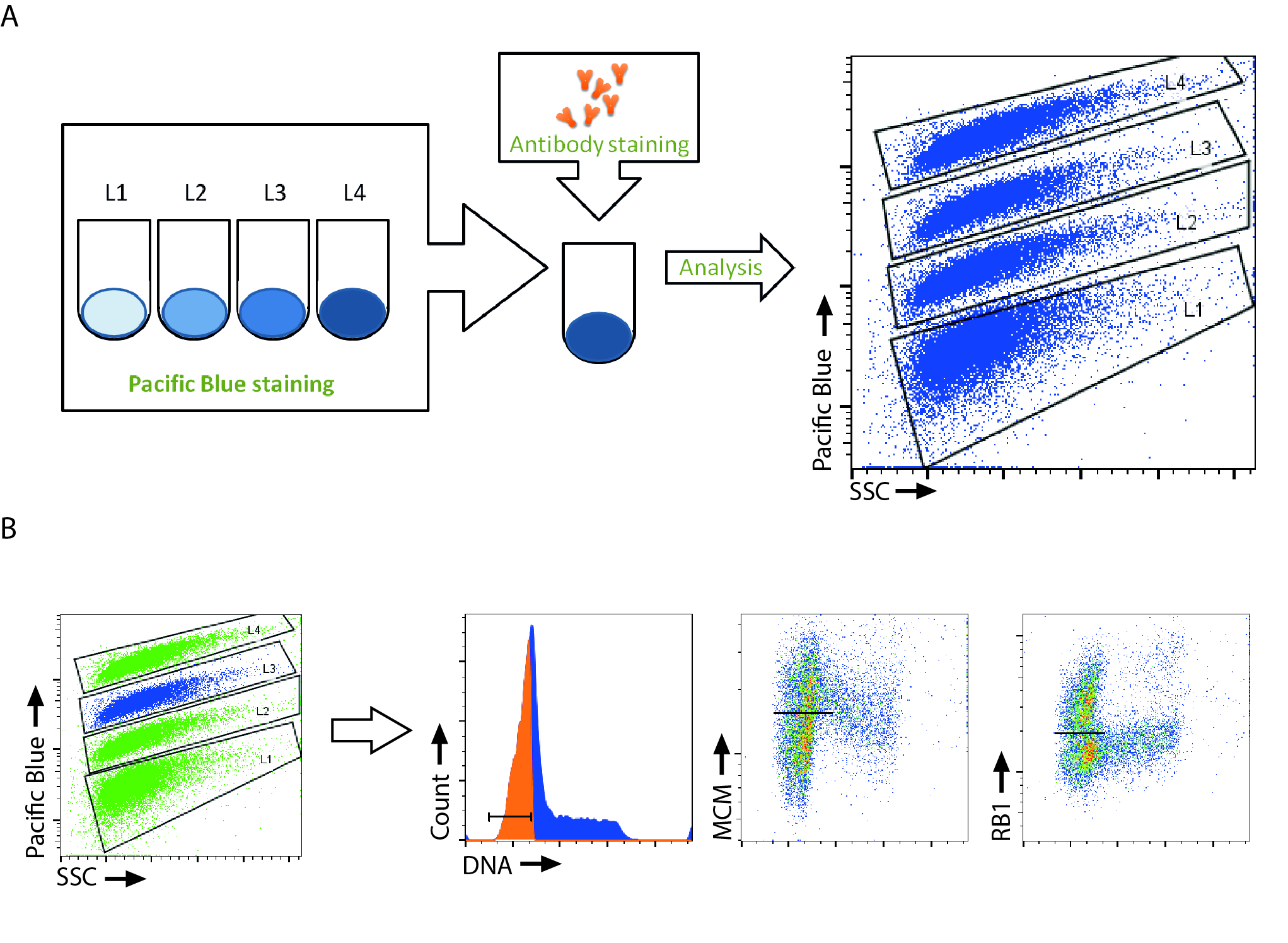

Supplement: SUPPLEMENTARY DATA [file supp_gkv744_nar-00925-met-g-2015-File009.tif]

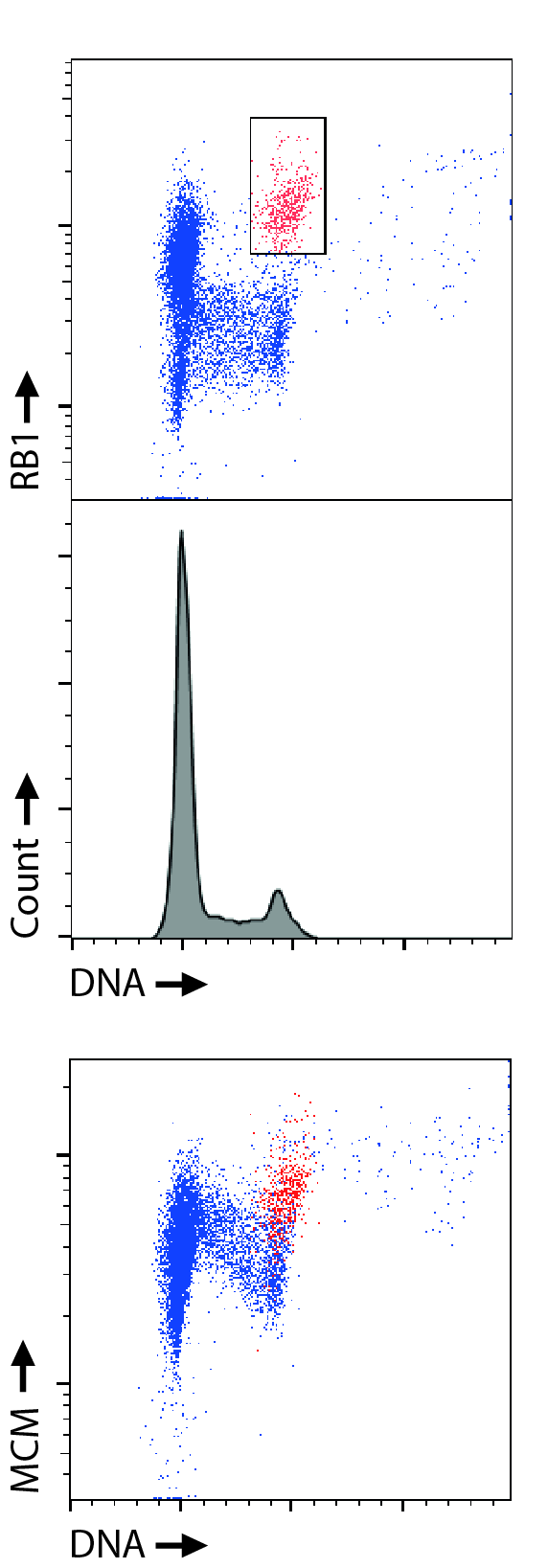

Supplement: SUPPLEMENTARY DATA [file supp_gkv744_nar-00925-met-g-2015-File010.tif]
